# Genetic profiling of human bone marrow mesenchymal stromal cells after *in vitro* expansion in clinical grade human platelet lysate

**DOI:** 10.3389/fbioe.2022.1008271

**Published:** 2022-10-12

**Authors:** Ann De Becker, Robbe Heestermans, Wouter De Brouwer, Kara Bockstaele, Ken Maes, Ivan Van Riet

**Affiliations:** ^1^ Department of Hematology, Universitair Ziekenhuis Brussel (UZ Brussel), Vrije Universiteit Brussel (VUB), Brussels, Belgium; ^2^ Research Group Hematology-Immunology, Vrije Universiteit Brussel (VUB), Brussels, Belgium; ^3^ Department of Clinical Biology, Universitair Ziekenhuis Brussel (UZ Brussel), Vrije Universiteit Brussel (VUB), Brussels, Belgium; ^4^ Clinical Sciences, Research Group Reproduction and Genetics, Centre for Medical Genetics, Universitair Ziekenhuis Brussel (UZ Brussel), Vrije Universiteit Brussel (VUB), Brussels, Belgium

**Keywords:** mesenchymal stromal cell, expansion, platelet lysate, gene expression, transformation

## Abstract

Mesenchymal stromal cells (MSCs) are non-hematopoietic cells that have a broad therapeutic potential. To obtain sufficient cells for clinical application, they must be expanded *ex vivo*. In the initial expansion protocols described, fetal calf serum (FCS) was used as the reference growth supplement, but more recently different groups started to replace FCS with platelet lysate (PL). We investigated in this study the impact of the culture supplement on gene expression of MSCs. Human bone marrow derived MSCs were expanded *in vitro* in FCS and PL supplemented medium. We found that MSCs expanded in PL-containing medium (PL-MSCs) express typical MSC immunomorphological features and can migrate, as their counterparts expanded in FCS-containing medium, through a layer of endothelial cells *in vitro.* Additionally, they show an increased proliferation rate compared to MSCs expanded in FCS medium (FCS-MSCs)*.* RNA sequencing performed for MSCs cultured in both types of expansion medium revealed a large impact of the choice of growth supplement on gene expression: 1974 genes were at least twofold up- or downregulated. We focused on impact of genes involved in apoptosis and senescence. Our data showed that PL-MSCs express more anti-apoptotic genes and FCS-MSCs more pro-apoptotic genes. FCS-MSCs showed upregulation of senescence-related genes after four passages whereas this was rarer in PL-MSCs at the same timepoint. Since PL-MSCs show higher proliferation rates and anti-apoptotic gene expression, they might acquire features that predispose them to malignant transformation. We screened 10 MSC samples expanded in PL-based medium for the presence of tumor-associated genetic variants using a 165 gene panel and detected only 21 different genetic variants. According to our analysis, none of these were established pathogenic mutations. Our data show that differences in culture conditions such as growth supplement have a significant impact on the gene expression profile of MSCs and favor the use of PL over FCS for expansion of MSCs.

## Introduction

Mesenchymal stromal cells (MSCs) are non-hematopoietic cells residing in a wide range of tissues such as the bone marrow, adipose tissue and umbilical cord (Friedenstein et al., 1974; Maqsood et al., 2020). There is a broad interest in the therapeutical use of these cells due to their biological characteristics. MSCs can be expanded *in vitro* at levels sufficient for clinical use. They have immunomodulatory capacities and can escape immune recognition since they do not express MHC class II antigens. Moreover, MSCs have a multipotent differentiation potential and can migrate to sites of tissue injury and inflammation (Majumdar et al., 2000; Vater et al., 2011; Li et al., 2013; Stagg and Galipeau, 2013). Tissue repair applications with these cells cover different domains of medicine, including orthopedics (Bashir et al., 2014), cardiology and neurology (Chen et al., 2001; Fukuda and Fujita, 2005). Other important applications, currently under investigation in hematology, are treatment and/or prevention of acute and chronic graft-versus-host disease (Le Blanc and Ringden, 2005) and support of engraftment after hematopoietic stem cell transplantation (HSCT) (Pinho and Frenette, 2019). Very recently it was found that MSCs can also be used for immunosuppressive therapy in patients with COVID-19 (Sengupta et al., 2020; Shu et al., 2020; Rezakhani et al., 2021). For clinical use, systemic delivery of exogenous MSCs is the preferred route of injection but this approach requires that the expanded MSCs have sufficient capacity to extravasate and home to the target tissue (Sarkar et al., 2011).

At this moment there is also increasing interest to investigate whether MSC-derived extracellular vesicles (EVs) such as exosomes, could serve as a cell-free biologic in clinical practice. MSCs exert their function at least partially through paracrine mechanisms (Konala et al., 2016). It can be assumed that EVs possess characteristics similar to their parent cells and might have some advantages over MSCs such as a better safety profile, easier maintenance and a smaller size enabling them to circulate over longer distance without entrapment in the lungs, a well-known problem when *in vitro* expanded MSCs are infused through the blood circulation (Gao et al., 2001).

Because of the large discrepancy between the number of cells that can be harvested *in vivo* and the number needed for clinical use, MSCs must be expanded *in vitro* (Wechsler et al., 2021). Until now, fetal calf serum (FCS) is used as the reference culture medium supplement for expanding MSCs (Friedenstein et al., 1974). However this supplement has several disadvantages, such as the possibility of transmission of bovine pathogens (prions, viruses, mycoplasmas and bacteria), a batch-to-batch quality inconsistency that hampers reproduction of results and ethical concerns regarding methods of FBS collection (Cholewa et al., 2011; De Becker and Van Riet, 2015; Burnouf et al., 2016). Several studies suggested that human platelet lysate (PL) might be a good alternative for FCS, providing some important advantages. PL has a more favorable safety profile whereas it is a humanized culture medium supplement, it induces a higher cell proliferation rate with a shorter time to reach culture confluency and it is readily available. In addition to these advantages, FCS and PL are made up of different components as indicated in Table 1. It is important to note that the list of components in this table is not exhaustive as the scientific community agrees that both supplements are still not fully characterized and show–sometimes important - batch-to-batch variability (Price and Gregory, 1982; Horn et al., 2010; Crespo-Diaz et al., 2011; Kinzebach et al., 2013; Shih and Burnouf, 2015; Pilgrim et al., 2022). When considering all these factors, it can be assumed that the expansion culture protocol, including the growth medium can have major influence on the biological MSC characteristics. Previous studies already demonstrated that *in vitro* expansion conditions can affect the phenotype of MSCs and influence their multipotency, DNA repair and proliferation regulation (Kretlow et al., 2008; Wagner et al., 2008). Therefore, further research optimizing the culture medium composition and expansion methods of MSCs is mandatory.

**TABLE 1:** Overview of the different components of FCS and PL as culture medium growth supplement. This list is non-exhaustive and based on previously published data (Price and Gregory, 1982; Horn et al., 2010; Crespo-Diaz et al., 2011; Kinzebach et al., 2013; Shih and Burnouf, 2015; Pilgrim et al., 2022). (bFGF = basis fibroblast growth factor, EGF = epidermal growth factor, HGF = hepatocyte growth factor, VEGF = vascular endothelial growth factor, PDGF = platelet derived growth factor, IGF = insulin like growth factor, CCL = chemokine (C-C motif) ligand, CXCL = C-X-C motif chemokine ligand).

Fetal calf serum	Human platelet lysate
Proteins e.g. hemoglobin, albumin, low antibody concentration	Proteins e.g. albumin, immunoglobulins, fibrinogen
Major cations: Na^+^, K^+^, Ca^++^	Coagulation factors
Major anions: Cl^−^, PO_4_^−^	Adhesion molecules
Essential amino-acids	Protease inhibitors and proteoglycans
Trace elements and vitamins e.g. Selenium, vitamin A, vitamin E	Growth factors e.g. bFGF, EGF, HGF, VEGF, PDGF-AA, PDGF-AB, PDGF-BB, IGF-1
Growth factors	Chemokines e.g. CCL5, CXCL1, CXCL2, CXCL3
Cytokines	Cytokines
Carbohydrates	
Hormones e.g. insulin, cortisol, parathyroid hormone, progesterone, testosterone, growth hormone, thyroid hormones	
Glucose	
Cholesterol Non protein nitrogen	

Based on clinical studies using *in vitro* expanded MSCs, it can be assumed that the cells are relatively safe in terms of genetic stability. To date, spontaneous transformation of human bone-marrow derived MSCs hasn’t been demonstrated yet (Barkholt et al., 2013; Budgude et al., 2020). Spontaneous transformation of MSCs has been observed so far only in expansion cultures using murine bone marrow cells. Aggressive sarcoma formation has been observed after transplantation of *in vitro* expanded murine MSCs in non-immunocompromised mice (Xu et al., 2012). These findings were supported by other studies (Tolar et al., 2007; Jeong et al., 2011; Xiao et al., 2013). The use of a medium supplement like PL that induces a higher proliferation rate, might be associated with a higher risk for genetic instability and malignant transformation.

In this study, we examined the impact of the culture medium supplement (PL *versus* FCS) on the genetic profile of *in vitro* expanded human mesenchymal stromal cells using genome wide RNA sequencing. In addition, we screened MSCs expanded in PL-based medium for the presence of tumor associated genetic variants by NGS using a targeted gene panel.

## Material and methods

### *In vitro* expansion of bone marrow-derived MSCs

Sternal bone marrow aspirates were obtained from healthy donors, following the informed consent. This study was approved by the Ethical Committee UZ Brussel (number 2001/23) For all donors MSCs were cultured in parallel in PL and FCS supplemented medium. The median age of the donors was 66 years (range 54–74 years) and 83% were male. Isolation of bone marrow mononucleated cells (BMMNC) was done by Lymphoprep density gradient centrifugation (Fresenius Kabi, Schelle, Belgium). BMMNC were plated in 10 ml DMEM +10% Hyclone FBS (Gibco, Fisher Scientific, Merelbeke, Belgium). According to the manufacturer, HyClone FBS is low in antibodies and high in growth factors to support robust growth and division of cells, including stem cells. In parallel, cells were cultured in α-MEM + 10% platelet lysate (Macopharma Benelux, Mouscron, Belgium) at a density of 60 × 10^6^ cells per 75 cm^2^ in Nunclon/Corning culture flasks (Nunc, VWR International, Leuven, Belgium) and incubated at 37°C with 5% humidified CO_2_. Growth medium and non-adherent cells were discarded after 4 h. The adherent cell fraction was rinsed with DPBS (Gibco) and 15 ml DMEM +10% FBS or α-MEM + 10% platelet lysate was added to the culture flask. Cells were cultured for four passages. For some experiments, cells were harvested after ten passages. At each passage, cells were detached after a 5 min Incubation at 37°C with trypsin 0.25% (Gibco). After adding 10% fetal calf serum (FCS) (A&E Scientific, Enghien, Belgium) in RPMI, the cells were washed once in DPBS (Gibco). After each passage, the cells were harvested when the cultures reached subconfluent conditions (50%–80% coverage of the culture surface area). The number of population doublings during *in vitro* expansion was calculated as previously described (Jung et al., 2012).

### Immunomorphological characterization of *in vitro* expanded MSCs

Approximately 10^5^ MSCs were incubated with 10 μl monoclonal antibody conjugated with fluorescein isothiocyanate (FITC) or phycoerythrin (PE): CD105-FITC (Biolegend, ImTec, Antwerpen, Belgium), CD90-PE (Biolegend), CD73-PE (Biolegend) and CD166-PE (Biolegend). Unbound antibody was washed after 15 min with 3 ml PBS (Gibco). The cell pellet was resuspended in 0.5 ml PBS (Gibco). Samples were analyzed with the flow cytometer Macs Quant (Miltenyi Biotec, Bergisch Gladbach, Germany) with 10,000 events recorded for each condition. The morphology of cultured cells was evaluated using light microscopy.

### *In vitro* migration of *in vitro* expanded MSCs

Prior to the migration assay, cells were labeled with the carbocyanine fluorochrome DiI (1,1′dioctadecyl-3,3,3′, 3′tetramethylindocarbocyanine perchlorate) (Molecular Probes, Fisher Scientific, Merelbeke, Belgium). DiI is a lipophilic molecule that incorporates in the cell membrane with specific spectral characteristics: absorption maximum at 549 nm and an emission maximum at 565 nm. MSC were incubated for 72 h with 10 μg/ml DiI at 37°C with 5% humidified CO_2_. After incubation, the cells were harvested with trypsin. Since MSCs were labeled with a fluorescent dye, we used BD Falcon™ HTS FluoroBlok Inserts (BD Benelux, Erembodegem, Belgium). Their polyethylene terephthalate (PET) membrane blocks light transmission from 490 to 700 nm. As such, we can detect cells present in the lower compartment only. Once cells migrate through the pores of the membrane, they are no longer shielded from the light source. From this moment, they can be detected with a fluorescence plate reader. Data were assembled with the Fluoroskan Ascent plate reader and software (Thermo-Labsystems, VWR International, Leuven, Belgium). Migration assays were performed using filters coated with 50 μg Matrigel (BD Benelux) (gelled at 37°C for 1 h) and human bone marrow-derived endothelial cells (4LHBMEC-line) to analyze transendothelial migration. MSCs (5 × 10^4^) in 100 μl RPMI were added to the upper compartment. The lower compartment contained 10% FCS in RPMI, which serves as a chemoattractant. Samples were incubated at 37°C overnight. The percentage migration was calculated as the ratio of signal intensity of migrated cells *versus* signal intensity of total input number of cells.

### Poly A + RNA sequencing

Bone marrow samples from four different donors were used to culture MSCs in both culture medium supplements (FCS *versus* PL) for four passages. After *in vitro* expansion, MSCs were harvested and RNA was isolated using the miRNeasy Micro Kit (Qiagen, Venlo, Netherlands) with column DNase digestion, following the manufacturer instructions. The RNA concentration was determined using the Qubit 2.0 Fluorometer (Thermo Fisher Scientific, Merelbeke, Belgium). RNA quality control was performed with the 2,100 Bioanalyzer microfluidic gel electrophoresis system (Agilent Technologies, Machelen, Belgium). The comparing RNA sequencing analyses was performed in collaboration with Biogazelle (Zwijnaarde, Belgium). Briefly, libraries for mRNA sequencing were prepared using the TruSeq stranded mRNA sample prep kit (Illumina Inc., San Diego, CA, United States). The starting 100 ng of RNA was mRNA enriched using the oligodT bead sytem (Illumina Inc.). Subsequently the isolated mRNA was enzymatically fragmented. Following to this, first and second strand synthesis were performed and the double stranded cDNA was purified with Agencourt AMPure XP (Beckman Coulter, Brea, CA, United States). The cDNA was end repaired and the fragment ends were ligated by illumine sequencing adaptors. The library was purified with Agencourt AMPure XP (Beckman Coulter). The polyA + RNA stranded libraries were pre-amplified with PCR and purified with Agencourt AMPure XP (Beckman Coulter). The size distribution of the libraries was validated and the quality inspected on the 2,100 Bioanalyzer high sensitivity DNA chip (Agilent Technologies). According to the project specification (number of reads), high quality libraries were quantified using the Qubit 2.0 Fluorometer (Thermo Fisher Scientific). The concentration was normalized and the samples were pooled. Single-end sequencing was performed on the NextSeq500 according to the manufacturer instructions (Illumina Inc.). Transcriptome data analysis based on eight samples with four in each group was based on the Tuxedo software package (Oracle Belgium, Vilvoorde, Belgium). The differentially expressed genes were finally organized in Gene Ontology categories using the open-source tool GOnet (Pomaznoy et al., 2018).

### Senescence analysis of expanded MSCs by β -galactosidase staining

Senescent cells within the MSC cultures were identified with a β-galactosidase staining using the Senescence Cells Histochemical Staining Kit (Merck Life Science, Hoeilaart, Belgium) according to the manufacturer’s instructions. MSCs of passage four were seeded in a 6-well plate at a density of 70.000 cells/well and FCS supplemented medium (Gibco) or PL supplemented medium (Macopharma Benelux) until reaching a sub confluent density. After addition of the staining mixture, the MSCs were incubated at 37°C without CO_2_ overnight. Stained MSCs were evaluated with light microscopy using the EVOS M7000 (Thermo Fisher Scientific).

### Detection of genetic variants by targeted gene sequencing

NGS analysis screening for genetic variants in 165 known tumor-associated genes was performed in collaboration with Brussels Interuniversity Genomics High Throughput core (BRIGHTcore), Universitair Ziekenhuis Brussel (UZ Brussel)/Vrije Universiteit Brussel (VUB), Brussels, Belgium. First, DNA was extracted from *in vitro* expanded MSCs using the QIAamp DNA Mini kit (Qiagen, Venlo, Netherlands), according to the instructions of the manufacturer. Eluted DNA was quantified on the Qubit 2.0 with the Qubit dsDNA HS Assay Kit (Life Technologies, CA, United States) after which DNA was stored at -20°C until library preparation. Next, DNA was requantified again using VICTOR Nivo^TM^(PerkinElmer, Waltham, United States) prior to library preparation. 150 ng of input DNA was used to generate libraries with the KAPA HyperPlus kit (Roche Sequencing, CA, United States). Target enrichment was performed according to version 5.0 of the manufacturer’s instructions with a homebrew Roche SeqCap EZ Choice probemix (Roche Sequencing, CA, United States) and homebrew IDT xGen Lockdown Probes for the final batch of sequenced samples (Integrated DNA Technologies, Inc. (IDT), Iowa, United States). A minimum of 14 million 2 × 100 bp reads were generated for each sample on the Illumina NovaSeq 6,000 system (Illumina Inc., CA, United States) using NovaSeq 6000 S2 Reagent Kit (200 cycles) kit. Illumina’s bcl2fastq algorithm (version 2.19) was used to convert the raw basecall files into fastq files after which reads were aligned to the human reference genome (hg19) using BWA (version 0.7.10-r789) and picard (version 1.97) was used to mark the duplicate reads. Genome Analysis Toolkit (GATK) (version 3.3) was used to provide post-processing of the aligned reads which consisted of realignment around insertions/deletions (indels) and base quality score recalibration. The quality control on the post-processed aligned reads was performed by using samtools flagstat (version 0.1–19) and picard HsMetrics (version 1.97) which were also used to investigate the total number of reads, the percentage of duplicate reads, the mean coverage on target and the percentage of on-target, near-target and off-target bases. Variants were called with GATK Mutect2 (version 4) in tumor-only mode and final variant files were described using Alamut batch version 1.11 and Alamut database version alamut_db-1.5-2021.06.01.db.

Filtering and interpretation of variants was performed based on the recommendations of the ComPerMed guidelines (version 2 January 2019) (Froyen et al., 2019) using an in-house-designed script with the exclusion of the following variants: known artefacts in Alamut, synonymous variants, variants with a MAF (minor allele frequency) of >0.1% and variants with a VAF (variant allele frequency) of <3%. After data filtering, the remaining variants are subjected to manual inspection of the aligned reads in IGV version 2.6.3 (Integrative Genomics Viewer, © Broad Institute and the Regents of the University of California). Biological significance of the detected variants and classification as “pathogenic,” “likely pathogenic” and “VUS” was assessed based on the recommendations described by Froyen et al. (Froyen et al., 2019).

## Results

### Culture and characterization of in vitro-expanded MSCs

**FIGURE 1:**
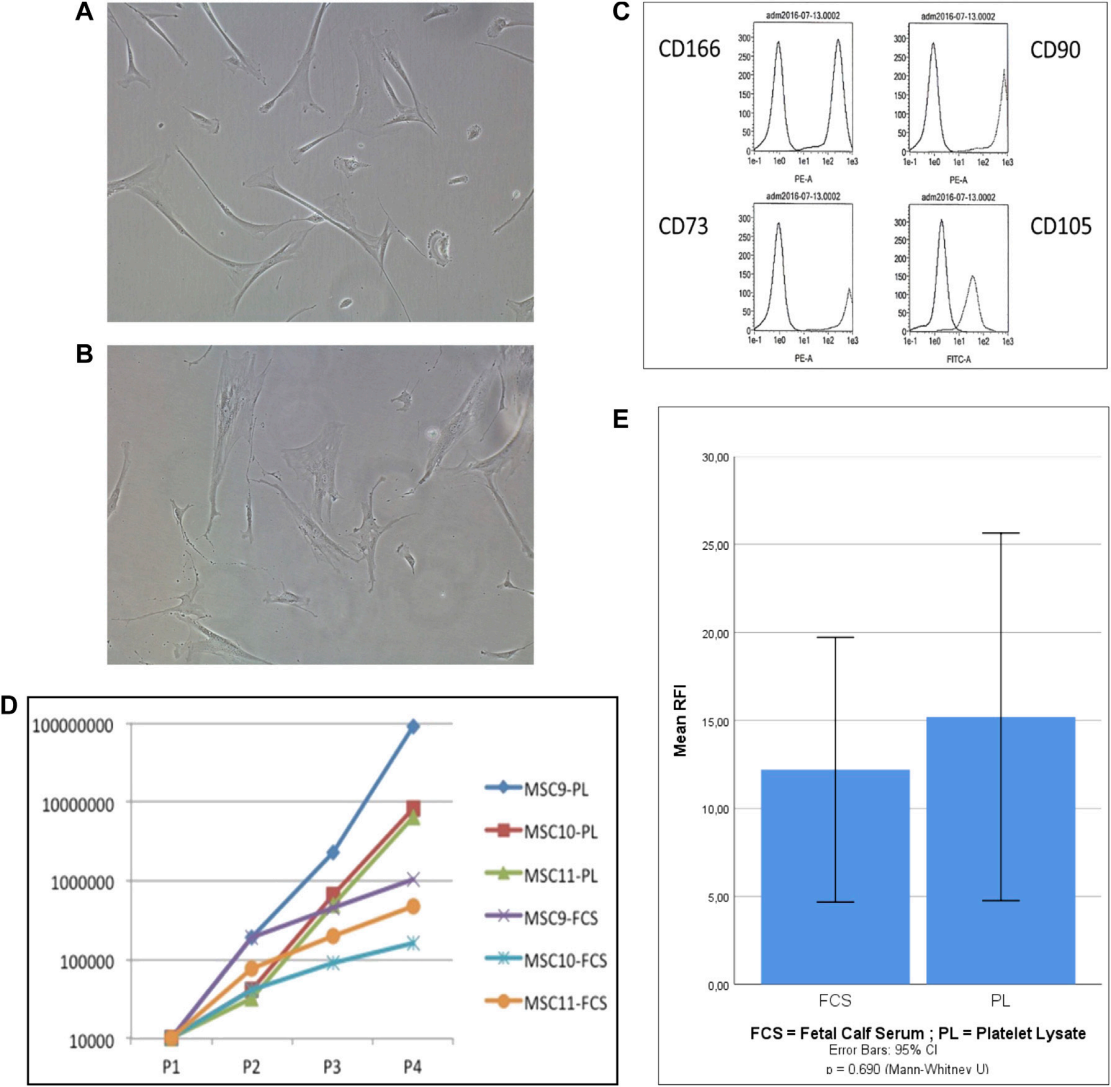
Characterisation of in vitro expanded MSCs. **(A,B)** Representative photographs of MSC’s cultured in PL-based **(A)** and FCS-based **(B)** expansion medium (30×); **(C)**: Phenotype MSCs expanded in PL-based medium: the first peak represents the negative control antibody whereas the second peak represents the antigen-specific antibody; **(D)**: Proliferation capacity of MSCs expanded in PL-based medium versus FCS-based medium (*n* = 3), the number of cells is presented according to the number of passages; **(E)**: *In vitro* migration of MSCs cultured in FCS-based medium and PL-based medium (*n* = 5). Migration is expressed as mean relative fluorescence intensity (RFI). (p= 0.69, 95% CI, Mann Whitney)*.*

Adherent cells were isolated by plastic adherence from mononuclear bone marrow cell suspensions and cultured in both types of expansion medium (PL-based *versus* FCS based) up to four passages (PL-MSCs and FCS-MSCs). For two donors, cells were cultured in PL-based medium up to 10 passages. FSC-MSCs showed a different shape and size compared to PL-MSCs (as shown in Figure 1). In PL-based medium (A) the cells showed in general a smaller cell size and had a more elongated (thinner) shape as compared to cells cultured in FCS-based medium (B). This morphology difference was inducible, as MSCs that were initially cultured in PL-based medium showed an increased cell size when they were passaged to FCS-based medium (data not shown). Flow cytometry analysis showed that the cells, expanded in both types of medium, had the same phenotype and expressed four markers: CD90, CD105, CD73, and CD166, known to be expressed by true MSCs (representative phenotype is shown in Figure 1C). The expansion rate of MSCs in PL-based medium was higher than in FCS-based medium, as shown for 3 donors in Figure 1D. Cells expanded in PL–based and FCS-based medium showed during the 4 passages a mean population doubling of 10.7 (±2.1) *versus* 5.4 (±1.3), respectively. As shown in Figure 1E, the percentage of migrated cells was compared between PL-MSCs and FCS-MSCs. Comparing the migration through the mimicked endothelium, PL-MSCs did not show a significantly increased migration as compared to MSCs cultured in FCS-based medium (*p* = 0.69, 95% CI, Mann Whitney).

### Differential gene expression of in vitro-expanded MSCs

**FIGURE 2:**
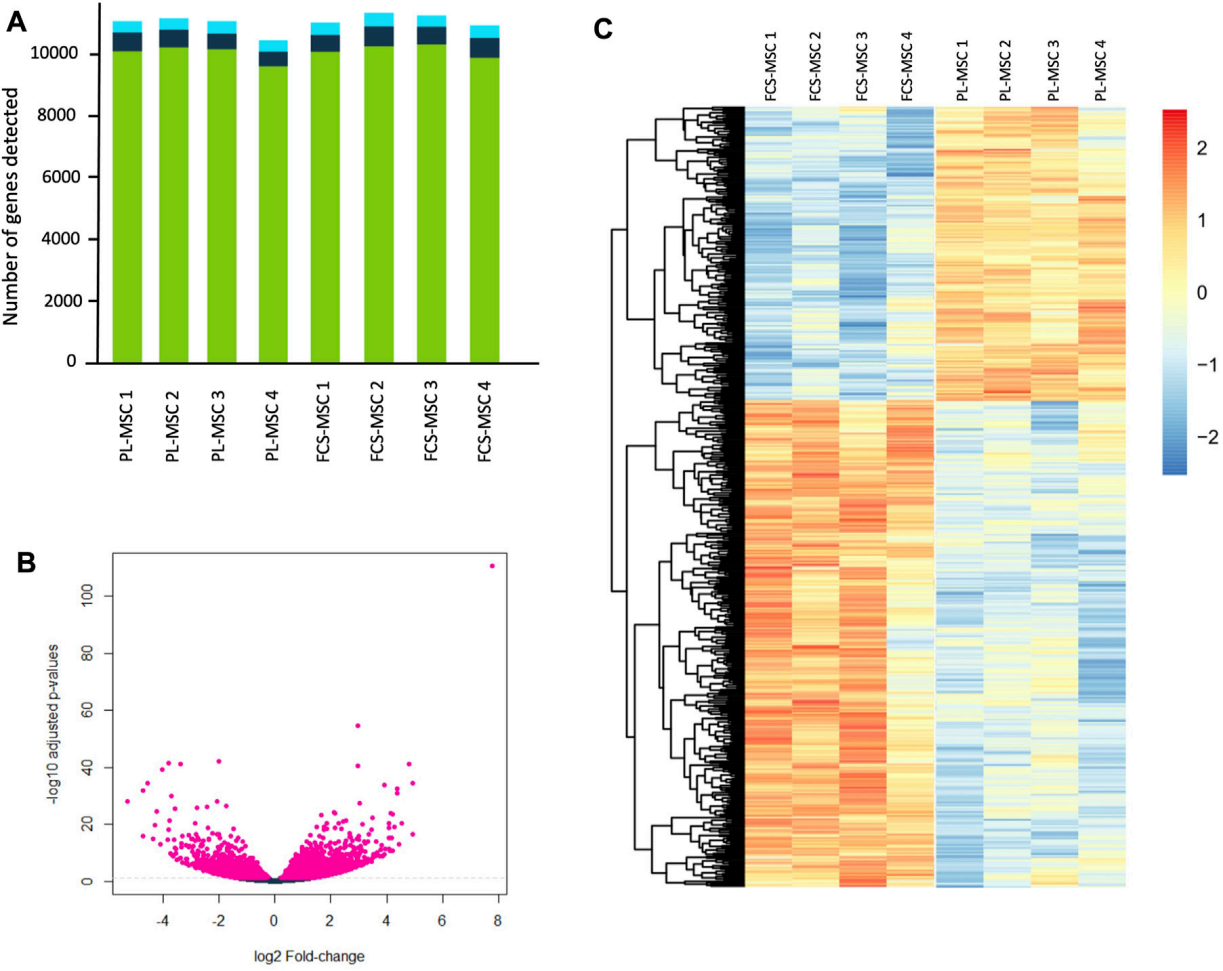
RNA sequencing of FCS-MSCs and PL-MSCs. **(A)**: Overview of the number of transcripts detected per sample for 4 different donors. The green bars represent coding genes, dark blue bars long non coding RNA and light blue bars other RNA species. **(B)**: Volcano plot showing -log10 adjusted *p*-values in function of the log2 fold change for FCS-MSCs *versus* PL-MSCs. Pink points indicate significantly differential expressed genes at FDR <0.05. FDR: false discovery rate. **(C)**: Heatmap showing the top 500 differentially expressed genes according to adjusted *p*-values at FDR <0.05 for FCS-MSCs *versus* PL-MSCs.

The RNA expression profile of MSCs cultured in FCS was compared to the cells cultured in clinical grade human PL. The data are available in the ArrayExpress repository. In total, 11,000 genes were evaluated (Figure 2A) of which **2,433** were upregulated in FCS and **2,260** in PL. We further narrowed the pool of genes and retained only those that were at least twofold up- or downregulated. This resulted in **1974** differentially expressed genes, **1,178** were upregulated in FCS and **796** in PL. Fragments per kilobase million (FPKM) normalizes the reads for read depth and gene length. When applying this additional filter (FPKM ≥ 0,3) we still retain **1,666** differentially expressed genes. This means that even with these more stringent criteria, still almost 1 in 6 genes is differentially expressed when a different growth supplement is used. A volcano plot depicts the differentially expressed genes with the log2 fold change value in the X axis and the -log10 adjusted *p*-value in the Y axis. This gives a quick overview of the number of significantly differentially expressed genes (Figure 2B). The top 500 differentially expressed genes are represented in the heatmap shown in Figure 2C. In the RNA sequencing dataset, we found that the 4 surface markers used to characterize MSCs are expressed at high levels in both cell types: mean FPKM ranging from 56 to 118 in FCS-MSCs and from 28 to 140 in PL-MSC. Interestingly, CD166 is significantly upregulated in FCS-MSCs according to the criteria described above: 2.45-fold change, *p*-value 3.57 × 10^−7^ and FPKM 80 in FCS-MSCs vs. 28 in PL-MSCs. CD90 on the other hand also has a *p*-value below 0.05 and FPKM 56 *versus* 100 but falls just short of the fold change of 2 criterium at -1.87 and is upregulated in PL-MSCs. CD73 and CD105 are not differentially expressed in this analysis.

**FIGURE 3:**
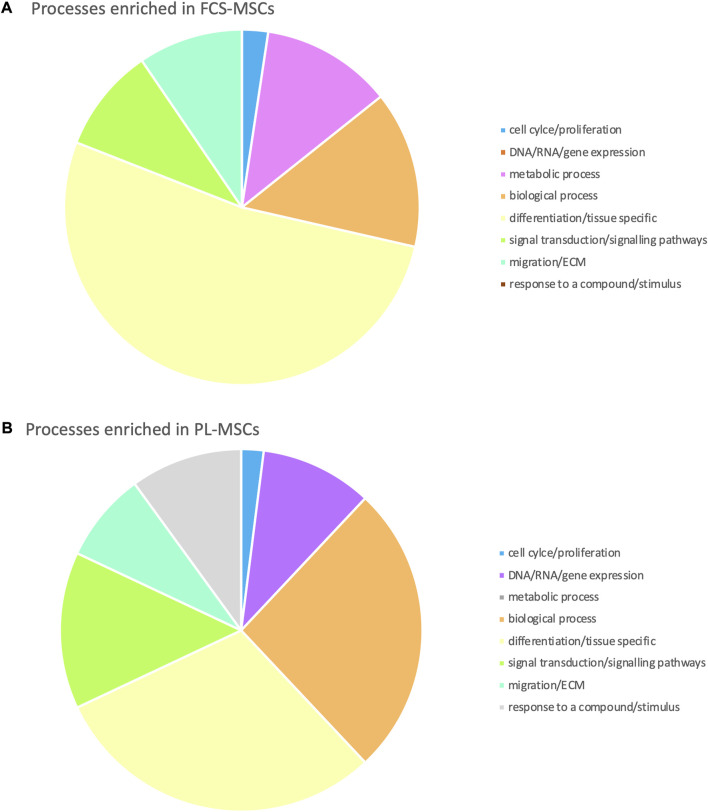
Gene Ontology analysis: processes enriched in **(A)** FCS-MSCs and **(B)** PL-MSCs (q ≤ 0,05, false discovery rate adjusted *p* value ≤ 0,05). Enriched processes are grouped in 8 major categories to get a better overview of processes impacted by differential expression due to use of a different growth supplement.

Using the online GOnet application enrichment for Gene Ontology (GO) terms was investigated. After selection of those GO terms that had a false discovery rate adjusted *p*-value of ≤0.05 we found that 92 GO terms were enriched: 42 for FCS-MSCs and 50 for PL-MSCs. We then organized these in 8 larger groups to reflect to which category of processes they belong (Figure 3). For both groups GO terms related to cell differentiation and biological processes made up more than half of enriched terms but differentiation and tissue specific GO terms were more prevalent in FCS-MSCs. Additionally, in PL-MSCs there is also enrichment of GO terms related to RNA/DNA/gene expression and responses to compounds or stimuli, these categories are not present in the GO analysis of FCS-MSCs.

Since PL-MSCs exhibit an increased proliferation rate we further analyzed the genes important for regulation of apoptosis and genes that play a role in cell senescence.

### Apoptosis

Table 2 summarizes the genes involved in the regulation of apoptosis. Three important anti-apoptosis genes show higher expression in MSCs cultured in clinical grade human PL-based medium. We see an almost 7 times higher expression of *BCL2A1* (BCL2 related protein A1) compared to FCS-based medium. *FAIM-2* (Fas-apoptotic inhibitory molecule 2) is almost 4 times higher expressed while *BCL-3* (B-Cell Lymphoma 3) is approximately 2 times upregulated in PL-based medium. *CASP1* (Caspase 1) is a pro-apoptotic gene upregulated in PL-MSCs. Genes involved in apoptotic processes and upregulated in MSCs, cultured in FCS-based medium, are *BCL-2* (BCL2 apoptosis regulator), *BMF* (BCL2 modifying factor), (each approximately 2 times higher) and *HRK* (Harakiri) (7 times higher). BMF and HRK are pro-apoptotic genes whereas BCL-2 is an anti-apoptotic gene. When also factoring in a minimum FPKM value of 0.3, FAIM2 and HRK can no longer be retained as significantly differentially expressed genes.

**TABLE 2:** Differential expression of genes involved in apoptosis in MSCs expanded in FCS and PL supplemented medium. Genes are designated by their gene name and expression data were filtered based on fold change (at least twofold) and *p*-value ≤0.05. To provide additional information on relevance of gene expression mean FPKM values for cells expanded in both conditions are also provided. (FPKM = fragments per kilobase million). The direction of differential expression is shown in the last column. These genes are at least twofold upregulated in FCS or PL.

Gene	Fold change	*p*-value	Mean FPKM FCS	Mean FPKM PL	Differential expression
BCL2A1	6.7×	8.02 × 10^−8^	0.25	2.28	Upregulated in PL
BCL3	2.2×	1.85 × 10^−3^	3.09	6.91	Upregulated in PL
CASP1	2.2×	2.08 × 10^−4^	0.75	1.58	Upregulated in PL
FAIM2	3.8×	1.68 × 10^−4^	0.028	0.21	Upregulated in PL
BCL2	2.2×	7.65 × 10^−3^	0.48	0.22	Upregulated in FCS
BMF	2.5×	2.09 × 10^−2^	0.36	0.09	Upregulated in FCS
HRK	7.1×	9.72 × 10^−6^	0.26	0.0086	Upregulated in FCS

**FIGURE 4:**
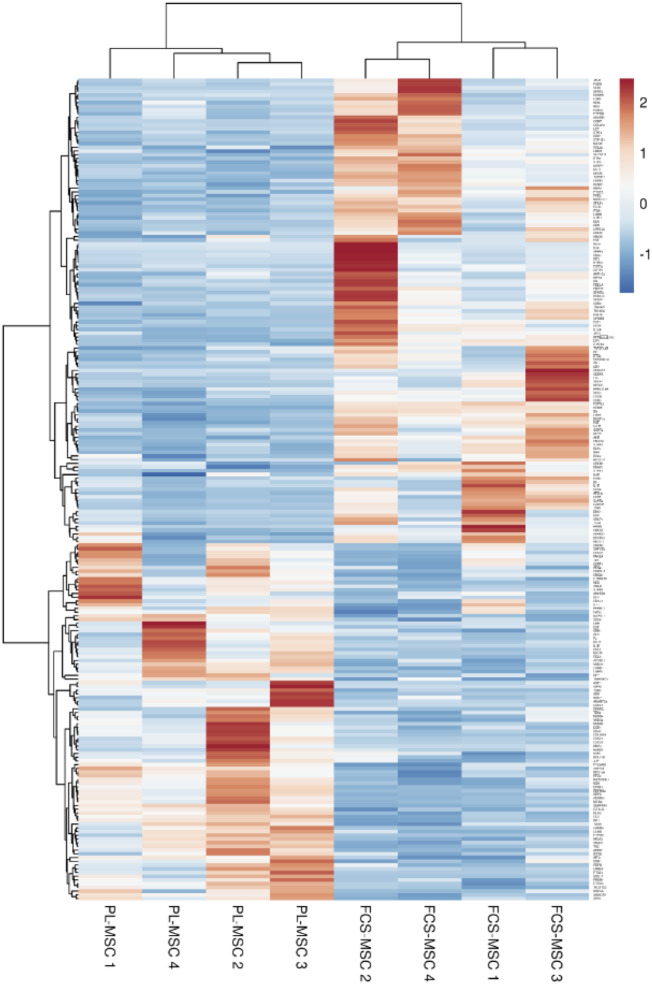
Heatmap generated based on FPKM of the 221 genes included in the Gene Ontology term ‘regulation of cell population proliferation’ (GO:004212). Rows are centered and unit variance scaling is applied to the rows. Both rows and columns are clustered using correlation distance and average linkage.

The Gene Ontology term ‘Regulation of cell population proliferation’ (GO:004212) was enriched in our RNA sequencing analysis. We selected the 221 genes included in this term and generated a heatmap using the Clustvis online tool (Metsalu and Vilo, 2015). In this analysis MSC show similar expression profiles according to the growth supplement used as evidenced by their clustering according to expansion in FCS or PL (Figure 4).

### Senescence of MSCs

**FIGURE 5:**
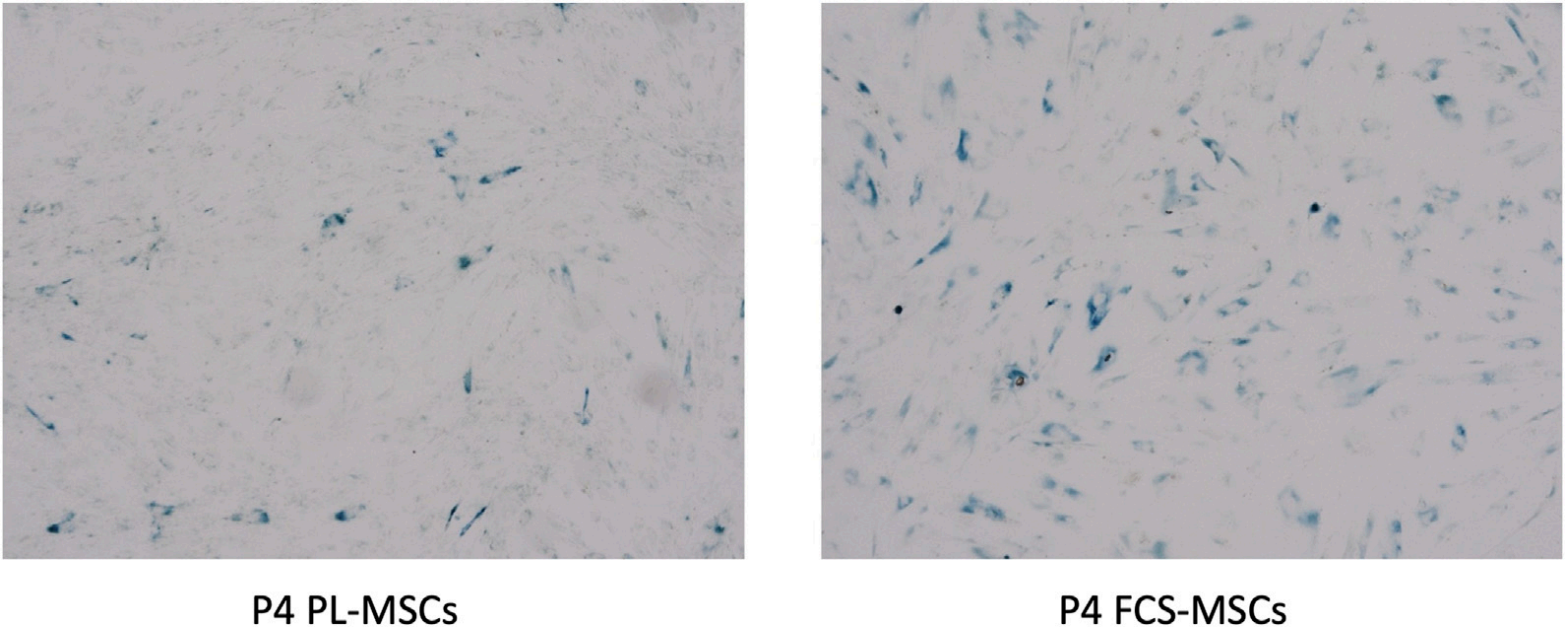
β-galactosidase staining of cultured MSCs. Cells from a representative donor were cultured in both media and stained after 4 passages. More β-galactosidase positive (blue colorored) cells were found in MSC cultures with FCS based expansion medium *versus* PL-based medium (4 × magnification) (P4 = passage 4).

In our hands FCS-MSCs show a blunted proliferation curve (Figure 1D) and cells become larger even at passage 4, PL-MSCs on the other hand do not appear to lose proliferation capacity at passage 4 and retain a small spindle shape morphology (Figures 1A,B). These might be signs of senescence in FCS-MSC. In 2008 a set of 33 genes that was differentially expressed in senescent MSCs was described. We cross-referenced this gene list with data obtained in our analysis (Tables 3, 4) (Wagner et al., 2008). We retrieved data on 20 genes reported upregulated in senescent MSCs. Of these 20 genes, 13 showed significant differential expression (*p* ≤ 0.05 and/or FPKM>0.3). Eleven genes are upregulated in FCS-MSCs, 2 in PL-MSC (Table 3). A few genes were also reported to be downregulated in senescent MSCs. In our analysis, only CXCL6 was significantly differentially expressed and was highly downregulated (38x) in MSCs expanded in FCS-medium (Table 4). By β-galactosidase staining it could be confirmed that more MSCs were in senescence modus when the cells were cultured in FCS based expansion medium *versus* PL based expansion medium (Figure 5).

**TABLE 3:** overview of differential expression of genes reported to be upregulated in senescent MSC passages. Genes are designated by their gene name. Fold change, *p*-value and FPKM for each gene are detailed in the columns. FPKM will be highest in the condition where the gene is upregulated. The direction of differential expression is shown in the last column. Genes that are considered not to be significantly up- or downregulated (*p* > 0.05 and/or FPKM <0.3) are marked in italic. (Gene list from Wagner et al., 2008).

Gene	Fold change	*p*-value	Mean FPKM FCS	Mean FPKM PL	Differential expression
GPNMB	11.3	3 × 10^−10^	93.8	4.16	Upregulated in FCS
MAN1C1	8.57	1.11 × 10^−11^	1.20	0.0821	Upregulated in FCS
*GCNT3*	*3.73*	*0.0066*	*0.0584*	*0.00341*	*Upregulated in FCS*
PERP	1.58	0.03	18.3	11.0	Upregulated in FCS
MCOLN3	7.46	1.5 × 10^−11^	1.09	0.0952	Upregulated in FCS
*ENPP5*	*1.39*	*0.27*	*1.88*	*1.296*	*Upregulated in FCS*
CTSK	3.63	0.0022	306	47.7	Upregulated in FCS
ATF3	−2.43	1.41 × 10^−6^	4.75	10.7	Downregulated in FCS
DAB2	1.59	0.044	39.9	24.8	Upregulated in FCS
LY96	1.50	0.045	20.7	12.7	Upregulated in FCS
DNAJB4	−1.70	0.035	26.0	50.0	Downregulated in FCS
SLC16A6	*1.67*	*0.20*	*2.44*	*1.10*	Upregulated in FCS
SLC11A2	*1.49*	*3.18 × 10* ^*−6*^	*5.96*	*3.80*	Upregulated in FCS
STAT1	2.22	4.3 × 10^−6^	100	41.5	Upregulated in FCS
*CA11*	*−1.46*	*0.44*	*0.0545*	*0.0654*	*Downregulated in FCS*
*SEC14L4*	*−1.24*	*0.72*	*8.82*	*6.56*	*Downregulated in FCS*
*SCG2*	*1.12*	*0.85*	*15.8*	*9.73*	*Upregulated in FCS*
GM2A	1.50	0.0090	1.28	1.30	Upregulated in FCS
*IFIT1*	*−1.06*	*0.80*	*110*	*101*	*Downregulated in FCS*
*PRNP*	*1.07*	*0.92*	*93.8*	*4.16*	*Upregulated in FCS*

**TABLE 4:** overview of differential expression of genes reported to be downregulated in senescent MSC passages. Genes are designated by their gene name in the first column. Fold change, *p*-value and FPKM for each gene are detailed in the columns. FPKM will be highest in the condition where the gene is upregulated. The direction of differential expression is shown in the last column. Genes considered not to be significantly up- or downregulated (*p* > 0.05 and/or FPKM <0.3) are marked in italic. (Gene list from Wagner et al., 2008).

Gene	Fold change	*p*-value	Mean FPKM FCS	Mean FPKM PL	Differential expression
CXCL6	−38.2	7.2 × 10^−29^	0.0405	6.53	Upregulated in FCS
*HAS1*	*1.87*	*0.059*	*33.7*	*14.3*	*Upregulated in FCS*
*RARRES1*	*−1.56*	*0.43*	*0.567*	*1.33*	*Upregulated in FCS*
*TNFSF11*	*0.90*	*0.87*	*0.196*	*0.338*	*Downregulated in FCS*

### Mutation profile of cultured MSCs

In the ten MSC DNA samples we investigated, 21 different genetic variants were detected as shown in Table 5, almost always with a high Variant Allele Frequency (VAF). Of these variants, only three are classified as “likely pathogenic” and these are located in *APC* (APC regulator of WNT signaling pathway), *NF1* (Neurofibromin 1) and *STAG2* (Stromal antigen 2). The remaining variants were either classified as VUS (17/21) or Ambiguous (1/21). The data are available in the ENA repository, accession number PRJEB55753.

**TABLE 5:** Overview of the genetic variants detected using a gene panel consisting of 165 tumor-associated genes. MSCs from donor 5 and 6 were harvested at passage 4 (P4) and 10 (P10) for sequencing, for all other donors cells were harvested at passage 4. VAF = variant allele frequency, VUS = variant of unknown significance.

Donor	Gene	cDNA	Protein change	VAF	Relevance of variant
1	*APC*	NM_000038.5:c.5427del	p. (Asp1810Ilefs*7)	4,1%	Likely pathogenic
	*NOTCH1*	NM_017617.4:c.4307C>T	p. (Ala1436Val)	49,4%	VUS
	*DIS3*	NM_014953.4:c.2646C>G	p. (Asn882Lys)	50%	VUS
	*RET*	NM_020975.4:c.364T>G	p. (Tyr122Asp)	57,8%	VUS
2	*RAD54L*	NM_003579.3:c.1784G>A	p. (Trp595*)	47,1%	Ambiguous
	*ATR*	NM_001184.4:c.3227A>G	p. (His1076Arg)	42,8%	VUS
	*WT1*	NM_024426.4:c.*10C>T	p. (?)	49,2%	VUS
	*ATM*	NM_000051.3:c.7114G>C	p. (Asp2372His)	51,5%	VUS
	*NF1*	NM_001042492.2:c.6904C>T	p. (Gln2302*)	36,8%	Likely pathogenic
3	*RAD54L*	NM_003579.3:c.883T>C	p. (Cys295Arg)	49,9%	VUS
	*PTPN11*	NM_002834.4:c.*13A>G	p. (?)	52,8%	VUS
4	*DICER1*	NM_030621.4:c.5738A>G	p. (Lys1913Arg)	48,2%	VUS
	*STAG2*	NM_001042749.2:c.3467 + 1_3467+3del	p. (?)	38%	Likely pathogenic
5 P4	*BRCA2*	NM_000059.3:c.3088T>G	p. (Phe1030Val)	50%	VUS
	*ERBB2*	NM_004448.3:c.1567C>T	p. (Pro523Ser)	49,5%	VUS
5 P10	*BRCA2*	NM_000059.3:c.3088T>G	p. (Phe1030Val)	49,6%	VUS
	*ERBB2*	NM_004448.3:c.1567C>T	p. (Pro523Ser)	48,6%	VUS
6 P4	*ERBB3*	NM_001982.3:c.2249G>A	p. (Arg750Gln)	48,9%	VUS
	*DNMT3A*	NM_175629.2:c.977G>A	p. (Arg326His)	3,6%	VUS
6 P10	*ERBB3*	NM_001982.3:c.2249G>A	p. (Arg750Gln)	52,9%	VUS
7	*ABL1*	NM_005157.5:c.2842C>T	p. (Pro948Ser)	52,5%	VUS
8	*ROS1*	NM_002944.2:c.2353G>A	p. (Val785Met)	50,5%	VUS
	*BRCA1*	NM_007294.3:c.4776C>G	p. (Asn1592Lys)	48,5%	VUS
	*GNA11*	NM_002067.5:c.911C>T	p. (Ala304Val)	47,7%	VUS

## Discussion

Initially, (FCS) was used as a growth supplement for culturing MSCs, and it has been considered a gold standard since then (Friedenstein et al., 1974). It is a rich source of many different growth factors and can act as a chelator for water insoluble nutrients, thus protecting the cells against shear damage (McGillicuddy et al., 2018). However, this medium has several disadvantages, such as xeno-immunization, pathogen transmission, batch-batch inconsistency but also ethical concerns (Cholewa et al., 2011; De Becker and Van Riet, 2015; Burnouf et al., 2016). Additionally bovine growth factors might not always be compatible with human cells, already in the early 80s Hornsby and colleagues found that for expansion of human adrenocortical cells, these responded better to horse than bovine serum (Hornsby et al., 1983). Around the same time Read et al. showed that bovine insulin-like growth factor-1 (IGF-1) has much lower affinity than human IGF-1 to the human receptor (Read et al., 1986). Human PL was proposed as a humanized alternative for FCS as a gold standard for the expansion of MSCs. This medium supplement has a more favorable safety profile since it is a humanized culture medium supplement and is readily available (Burnouf et al., 2016). In wound healing degranulation of platelets leads to secretion of cytokine and growth factors such as epidermal growth factor, PDGF, insuline like growth factor 1 and TGFβ1 and TGFβ2. These factors are important to start different aspects of wound healing (Martin P, 1997). In 2008 Ng and colleagues showed that platelet derived growth factor (PDGF), transforming growth factor β (TGFβ) and fibroblast growth factor (FGF) are important for proliferation and differentiation of MSCs (Ng et al., 2008). These growth factors appear to be present in abundance in PL, that is often generated by repeated cycles of freezing and thawing. Moreover, proteomics and cytokine array assays have confirmed the importance of platelet derived factors for *ex vivo* expansion of MSCs (Horn et al., 2010; Kinzebach et al., 2013). Additionally, it has also been shown that of all platelet derivatives that can be used for expansion of MSCs, only PL will affect the MSC proliferation rate (Bieback et al., 2009).

In this study we aimed to investigate the impact of culture conditions such as the choice of growth supplement on the genetic profile of MSCs.

Firstly, we characterized PL-MSCs expanded according to our protocol in a culture medium supplemented with clinical grade PL. These cells showed the typical spindle shaped morphology of MSCs and expressed CD73, CD90, CD105 and CD166 on the cell surface. Next MSCs from the same donor were cultured in parallel in both FCS and PL supplemented culture media. We could see that PL-MSCs have a higher proliferation rate than those cultured in FCS. *In vitro* transendothelial migration assays show a similar migration capacity *in vitro* for PL-MSCs and FCS-MSCs.

Changes in biological characteristics and gene expression profile in MSCs after alterations in the culture conditions have been reported previously (De Becker et al., 2007; Shi et al., 2007). Not just culture conditions but also the source used to obtain MSCs can have a significant impact on MSC gene expression profile and biological characteristics. Adipose tissue MSCs for example have less potential for bone formation and this is reflected by upregulation of genes that impact adipogenic but not osteocytic differentiation in these MSCs (Gluscevic et al., 2020). We hypothesized that the choice of growth supplement for *ex vivo* expansion of MSCs also might influence gene expression of these cells. Therefore, we performed whole genome polyA + RNA expression profiling on bone marrow derived MSCs from different donors, cultured in parallel in FCS and PL supplemented medium. These analyses provided evidence that the gene expression profile changes considerably when a different growth supplement is used. Of 30,000 genes evaluated almost 4,700 were differentially expressed. Taking only those genes into account with at least a twofold change, *p*-value ≤0.05 and FPKM ≥0.3, still 1,666 genes were differentially expressed of which 973 were upregulated in FCS-MSCs and 693 in PL-MSCs. We found that of the 4 typical surface markers used to characterize MSCs, CD166 is significantly upregulated in FCS-MSCs and CD90 falls just short of the 2-fold change criterium but has a very low *p*-value and high FPKM suggesting that these data are robust. It has been shown previously that different conditions such as proliferative state, serum deprivation or cryopreservation can change the expression of surface antigens (Dudakovic et al., 2014; Camilleri et al., 2016; Krull et al., 2021). In future applications or design of quality control assays for release of MSC products, differential expression of some surface markers that reflect for example a post-proliferative state might be of interest.

To gain more insight in the function/role of all these genes, Gene Ontology analysis was performed using an online tool (Pomaznoy et al., 2018). This analysis shows that different processes are enriched in MSCs if cultured in FCS or PL. In FCS-MSCs 42 GO-terms are enriched, most of these are related to tissue specific processes and differentiation. In PL-MSCs 50 GO terms are enriched, in these cells differentiation/tissue specific and biological processes account for just over half of all GO terms enriched. In PL-MSCs terms related to RNA/DNA and gene expression and response to a compound/stimulus are also enriched which is not the case in FCS-MSCs. This type of analysis provides an enormous amount of information that should be interpreted carefully, for example in MSCs expanded with PL medium more genes could be upregulated in GO processes of migration, but this does not automatically translate into an improved migration capacity. The upregulated genes might also be inhibitory molecules. To clear this out the upregulated genes and their functions must be identified. In our study we are confronted with a very high number of differentially expressed genes and it is beyond the scope of this work to catalogue all. Since we–and others before us–observed an increased proliferation capacity of PL-MSCs we focused on differential expression of genes involved in apoptosis, proliferation and senescence as will be discussed further (Salvadè et al., 2010; Luzzani et al., 2015).

We identified 7 apoptosis related genes in our analysis. Of these 4 were upregulated in PL-MSCs and 3 in FCS-MSCs. Three of 4 genes upregulated in PL-MSCs are anti-apoptotic (BCL2A1, BCL-3, FAIM-2) whereas in FCS supplemented MSC 2 of 3 upregulated genes are pro-apoptotic (BMF, HRK). After more stringent filtering, including a FPKM threshold of 0.3 we find that FAIM2 in PL-MSCs and HRK in FCS-MSCs cannot be considered significantly upregulated in the respective culture conditions. This shows that we should interpret these data with caution, FPKM values are low in both conditions, signaling low gene expression. BCL2A1, a member of the BLC-2 protein family, expression is 6.7 times higher after expansion in PL containing medium. It forms hetero- or homodimers and acts as an anti-apoptotic regulator in a wide range of cellular processes. The protein encoded by this gene blocks the activation of caspases and reduces the release of pro-apoptotic cytochrome c from the mitochondria (Flores-Romero et al., 2019). BCL-3 is also upregulated after culture in PL, this molecule is involved in cell cycle regulation, plays a role in maintaining pluripotency of embryonic stem cells and is anti-apoptotic (Flores-Romero et al., 2019; Liu et al., 2022). The pro-apoptotic gene upregulated in PL-MSCs is CASP-1, a member of the caspase family which plays an important role in the execution of cell death (Valenti et al., 2021). BMF is upregulated in FCS-MSCs and is important for triggering apoptosis in response to intracellular damage (Piñon et al., 2009). BCL-2 is the anti-apoptotic gene upregulated in FCS-MSCs. This gene encodes a protein located on the outer mitochondrial membrane that blocks apoptosis of, among others, lymphocytes (Bruckheimer et al., 1998). These observations show differential expression of genes involved in apoptosis and perhaps suggest a different gene expression profile of proliferation associated genes. After all, we observed a number of population doublings in PL-MSC cultures that is approximately twice as high as in FCS-MSC cultures. Other groups have shown earlier that proliferation rate of MSCs in PL supplemented medium appears higher than in FCS supplemented medium (Horn et al., 2010; Crespo-Diaz et al., 2011; Kinzebach et al., 2013). As stated above RNA sequencing and subsequent GO analysis gives an abundance of information but in many processes large numbers of genes are involved. To get a visually informative overview of the differential expression of these genes a heatmap is a useful tool. Observing an increased proliferation potential and upregulation of 2 anti-apoptotic genes with higher FPKM in PL-MSCs and enrichment of the GO term ‘Regulation of cell population proliferation’ we created such a heatmap for these genes. This heatmap shows a clustering of MSCs according to growth supplement, confirming an effect of these supplements on expression of genes relevant for proliferation. A concern might be that differences in culture confluence levels influence expression of genes involved in proliferation as confluent cells will stop dividing. We harvested our cells at similar levels of subconfluency and thereby hope to minimize this possible bias in our analysis. Camilleri et al. showed that confluent and proliferating MSCs show different gene expression profiles. They identified CD168 as a gene exclusively expressed in proliferating cells and CD106 as a gene only expressed in confluent MSCs. In the samples we included in this analysis there was no difference in expression of both genes (data not shown).

Wagner and colleagues described the effects of senescence on MSCs. Prolonged passaging, up to 12 passages, resulted in morphological changes, the cells became larger and ultimately stopped to proliferate. At different passages they performed mRNA profiling. Based on these analyses they identified 29 genes that were significantly upregulated and 4 genes that were significantly downregulated in senescent passage MSCs (Wagner et al., 2008). Since we observed a higher proliferation rate in PL-MSCs but found the morphological aspect of senescence rather in FCS-MSCs (cells were larger), we evaluated expression of these genes in our RNA sequencing analysis comparing PL to FCS. In total 20 genes listed in the paper of Wagner were also included in our analysis, 65% (13/20) of genes that were upregulated in their analysis are also significantly differentially expressed in our analysis comparing FCS and PL as growth supplements. Of these 86% are upregulated in MSCs cultured in FCS-based medium ranging from 1.5–11 times. Wagner et al. also reported 4 genes that were downregulated (Wagner et al., 2008). In our analysis only 1 gene is statistically significant differentially expressed: CXCL6, it is highly downregulated (38 times) in FCS-MSCs. Based on these observations we can conclude that the gene expression profile of FCS-MSCs shows signs of senescence. Some genes were also upregulated in PL-MSCs but a much smaller number and to a smaller degree (1.7 and 2.4 fold). Beta galactosidase staining of cells is a well-established assay to screen for senescent cells. Using this technique we found more senescent cells in passage 4 FCS-MSCs than in passage 4 PL-MSCs, confirming our observations in the RNAseq analysis. Based on these results we favor the use of PL-MSCs for the *in vitro* expansion of MSCs. After all, senescent MSCs have diminished proliferative, differentiation and immunomodulatory capacities. They also appear to be less able to support hematopoiesis (Wagner et al., 2008; De Witte et al., 2017; Gnani et al., 2019).

To obtain a sufficient cell number for therapeutic applications MSCs are expanded *in vitro*. We have shown that especially in PL supplemented medium, MSCs can grow rapidly, and RNA profiling showed upregulation of anti-apoptotic genes. The last part of this study focuses therefore on the risk of malignant transformation MSCs expanded in PL *in vitro*. To screen for genetic changes that could lead to malignant transformation, we used a state-of-the art next generation sequencing approach to characterize the mutation profile of the coding DNA sequences of these MSCs in both low and high passages. Based on this analysis, only 21 different genetic variants in total were detected (after data filtering) in the 10 MSC DNA samples that have been sequenced. The majority (17/21, 81%) of these variants constituted of VUS (variants of uncertain significance). As the name suggests, these are all variants of which the biological significance is unclear, and where the currently available evidence is certainly insufficient to assume a potential pathogenic effect of these variants. Only three variants were detected that could be classified as “likely pathogenic” (in genes *APC*, *NF1,* and *STAG2*), meaning that there are reasons to assume a potential harmful effect of these variants but there is no consensus nor sufficient (functional) evidence to confirm this pathogenicity. Genetic variants in *APC* have been implicated in familial adenomatous polyposis (FAP) and colorectal cancer (Fodde R, 2002). The *APC* p.Asp1810Ilefs*7 variant had a low VAF (4,1%), indicating that not all cells carried the mutation and it does not appear to induce an immediate advantage for expansion *in vitro*. Similarly, although the *NF1* p.Gln2302* variant and the *STAG2* splice site variant we found were classified as likely pathogenic, no compelling evidence of a pathogenic functional effect of these particular variants could be retrieved in literature. Interestingly, based on a review of the clinical data from the donors, we found antecedents of colon polyps in the donor in whom the *APC* variant was found, although we did not detect the typical *APC* mutations seen in FAP (Fodde R, 2002). The donor in whom the *NF1* variant was detected did not show any familial or personal antecedents of neurofibromatosis but had antecedents of diabetes and psoriasis which implies a chronic state of inflammation and thus an increased risk of developing acquired mutations (Lonkar and Dedon 2011). The same observation was made in donor 4 who had a *STAG2* variant and suffered from COPD which is again a chronic state of inflammation. The cells in these donors have therefore had ample opportunity to acquire genetic variants *in vivo*. A baseline analysis of the BM sample at the start of the cell culture could give a definitive answer to the question whether these mutations discussed above were indeed pre-existing.

Importantly, we observed no major changes in the mutation signature of the donors between passage 4 and passage 10, as can be derived from Table 4. The fact that no additional (pathogenic) variants emerged in P10 provides further evidence and reassurance that no major genetic changes during *in vitro* expansion of MSCs occur that may lead to malignant transformation. When taking these observations into account together with the number of population doublings, these results indicate that at least the exonic (and clinically most important) DNA sequences remain largely stable during *in vitro* expansion of MSCs, supporting the safe use of PL for *in vitro* expansion of MSCs. In clinical trials with human MSCs there have been no reports of malignant transformation so far (De Becker and Van Riet 2015). However, murine MSCs have been shown to be prone to malignant transformation after prolonged *in vitro* expansion and Sole et al. were able to generate malignant cells *in vitro* from MSCs derived from an Ewing sarcoma patient (Xu et al., 2012; Sole et al., 2021). Kim et al. performed whole genome sequencing of MSCs at different passages and warned for genomic instability of human MSCs in case of prolonged *ex vivo* expansion (Kim et al., 2017). Additionally, MSCs derived from patients with myeloid malignancies were reported to have cytogenic abnormalities or genetic variations (Kouvidi et al., 2016; Azuma et al., 2017). Our data suggest that human bone marrow derived MSCs expanded in PL supplemented medium are safe and unlikely to undergo malignant transformation after administration.

The data obtained in this study lead us to conclude that the selection of expansion medium can have a significant impact on the biological characteristics and gene expression profile of *in vitro* expanded human bone marrow derived MSCs. The option to replace the standard FCS-based medium by human platelet lysate–based medium for clinical grade MSC expansion seems to be advantageous: the cells retain MSC characteristics and exhibit a higher proliferation capacity without showing signs of senescence. FCS-MSCs on the other hand show signs of senescence already at passage 4 in our hands. Taking the higher *in vitro* proliferation rate of PL-MSCS into account, we could not provide any evidence for an increased risk of malignant transformation in the expanded MSCs. Future research should validate the biological effects of this human growth supplement as well as its potential to improve the clinical use of *in vitro* MSCs and their derived therapeutic products.

## Data Availability

The original contributions presented in the study are publicly available. NGS data set can be found in the ENA Repository https://www.ebi.ac.uk/ena/browser/ (accession number PRJEB55753). RNA seq data set can be found in ArrayExpress https://www.ebi.ac.uk/arrayexpress/ (accession number E-MTAB-12272).
